# Atomically Thin B doped g-C_3_N_4_ Nanosheets: High-Temperature Ferromagnetism and calculated Half-Metallicity

**DOI:** 10.1038/srep35768

**Published:** 2016-10-20

**Authors:** Daqiang Gao, Yonggang Liu, Peitao Liu, Mingsu Si, Desheng Xue

**Affiliations:** 1 Key Laboratory for Magnetism and Magnetic Materials of MOE, Key Laboratory of Special Function Materials and Structure Design, Ministry of Education, Lanzhou University, Lanzhou 730000, People’s Republic of China

## Abstract

Since the graphitic carbon nitride (g-C_4_N_3_), which can be seen as C-doped graphitic-C_3_N_4_ (g-C_3_N_4_), was reported to display ferromagnetic ground state and intrinsic half-metallicity (Du *et al*., *PRL*,*108*,*197207*,*2012*), it has attracted numerous research interest to tune the electronic structure and magnetic properties of g-C_3_N_4_ due to their potential applications in spintronic devices. In this paper, we reported the experimentally achieving of high temperature ferromagnetism in metal-free ultrathin g-C_3_N_4_ nanosheets by introducing of B atoms. Further, first-principles calculation results revealed that the current flow in such a system was fully spin-polarized and the magnetic moment was mainly attributed to the *p* orbital of N atoms in B doped g-C_3_N_4_ monolayer, giving the theoretic evidence of the ferromagnetism and half-metallicity. Our finding provided a new perspective for B doped g-C_3_N_4_ spintronic devices in future.

Recent spintronic devices are proposed to simultaneously utilize the charge and spin freedom of electron for logic and memory devices, which ignited a revolution in information processing for the electronic devices^1^,^2^. To achieve this goal, injecting spin into the electronic materials is necessary and searching for the materials which can generate 100% spin-polarized currents at the Fermi level is the urgent issue. Half-metal materials can filter the current into a single spin channel without any external operation, fully meet this demand and play an essential role in the spin-dependent transport study^3^,^4^,^5^,^6^. Motivated by this trend, lots of transition-metal-contained materials showing half-metallicity were investigated in the past decades, such as CoS_2_^7^, MoN_2_^8^, Cu doped ZnO^9^ and Cd_1−x_Cr_x_Z (Z = S, Se and Te) dilute magnetic semiconductor compounds^10^, providing their potential applications in the spintronic devices. However, most of the transition-metal-contained half-metal materials may not be compatible with present mature technologies, as well as, the heavy transition-metals are generally toxic for biological systems.

Recently, the appearance of half-metallic ferromagnetism in edge-modified zigzag graphene nanoribbon opened another exciting pathway for the development of next generation metal-free spintronics^11^. Generally speaking, a long spin relaxation time is an important factor for spin information delivery in the spin-dependent transport study, and the ferromagnetic two-dimensional (2D) materials with magnetism arising from pure *s/p* electrons present the weak spin-orbit coupling. By virtue of the fact that the spin relaxation time is inversely proportional to the strength of spin−orbit coupling, the half-metallicity ferromagnetism in the 2*p* electron system will bring an interesting issue in the spin-dependent transport property, which is more suitable for the future generation of spintronic devices^12^,^13^. Therefore, exploiting half metallic ferromagnetism in *s/p* electron systems is a desired goal in recent researches. Although ideal graphene and graphite are non-magnetic in nature, the presence of point defects and zigzag edge in graphitic networks may induce spin polarization and lead to the formation of local magnetic moment. Besides, the isostructural hexagonal boron nitride (BN) and ternary B-C-N systems are also intriguing candidates as metal-free spintronic devices due to their reported ferromagnetism and half metallicty^14^,^15^,^16^,^17^,^18^.

Recently, Du *et al*. revealed that the graphitic carbon nitride (g-C_4_N_3_), which can be seen as C-doped graphitic-C_3_N_4_ (g-C_3_N_4_), displayed a ferromagnetic ground state and an intrinsic half-metallicity^19^. This result immediately attracted a great deal of research interest, given that it demonstrates a hitherto efficient route to tune the electronic structure and magnetic properties of g-C_3_N_4_. For examples, Xu *et al*. and our previous results indicated that g-C_3_N_4_ ultrathin nanosheets with hydrogen dangling bonds and fluoride adsorption showed obvious room temperature ferromagnetic behavior, at the same time, the observed ferromagnetism could even be tuned by the concentration of adatoms^20^,^21^. Most recently, Meng *et al*. also concluded that the triazine-based g-C_3_N_4_ monolayer doped with elements of B at the center of the triazine ring showed half-metallicity ferromagnetism order. In particular, the estimated Curie temperature of 1200 K made it an excellent potential candidate for spintronics applications in future^22^, but there has been no direct experimental evidence thus far. Further, owing to its stable structure of coplanar tri-s-triazine based g-C_3_N_4_ than that of triazine-based one, it is necessary to study the electronic structure and magnetic properties of B atom doped g-C_3_N_4_ in tri-s-triazine based structure. In this paper, ultrathin B-doped g-C_3_N_4_ nanosheets with tri-s-triazine structure were prepared by heating the mixture of melamine and boron oxide in the closed system with N_2_ atmosphere and post-ultrasound exfoliation method^23^. Results indicated that the atomic-thick B-doped g-C_3_N_4_ nanosheets exhibit intrinsic ferromagnetism, as well as, possible half metallic, which is also confirmed by theoretical calculations. This work opens a new door in the search for other metal free half-metallic ferromagnetic systems.

## Results

**Figure 1:**
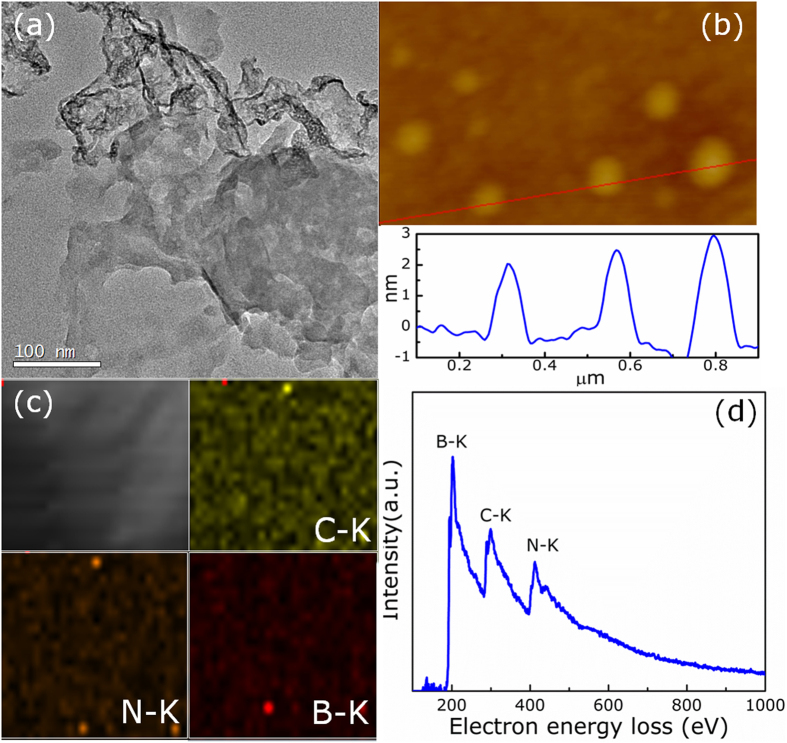
(**a**) TEM image, (**b**) AFM image and the corresponding height profile, (**c**) The elemental mapping and (**d**) EELS spectrum for B doped g-C_3_N_4_ nanosheets.

X-ray diffraction analysis (Fig. S1) reveals the graphite like packing of all products, showing the typical (002) interlayer-stacking peak around 27.4°, which is similar that other reported g-C_3_N_4_ with tri-s-triazine structure^24^. As can be seen that the (002) peak becomes broader and gradually less intense with increasing the concentration of B, causing by the disturbance of graphitic structure by B atoms introduction. Figure 1a shows the transmission electron microscopy (TEM) image of the product, revealing a nanosheet feature. The thickness of the as-obtained B-doped g-C_3_N_4_ nanosheets were measured by tapping-mode atomic force microscopy (AFM), as shown in Fig. 1b, where the thickness of the nanosheets is estimated to be 2~3 nm. To identify the elemental composition of B-doped g-C_3_N_4_ nanosheets, we further investigated the sample by elemental mappings and electron energy loss spectroscopy (EELS). As shown in Fig. 1c, results present the elemental mappings of B, N, and C, which confirms homogeneous distributions of B, N, and C elements on the nanosheets. Besides, the corresponding EELS spectrum shows the core-loss K-edge of B, N, and C located at 189, 398, and 686 eV, respectively (Fig. 1d), indicating B element was introduced in the g-C_3_N_4_ frame^25^,^26^.

**Figure 2:**
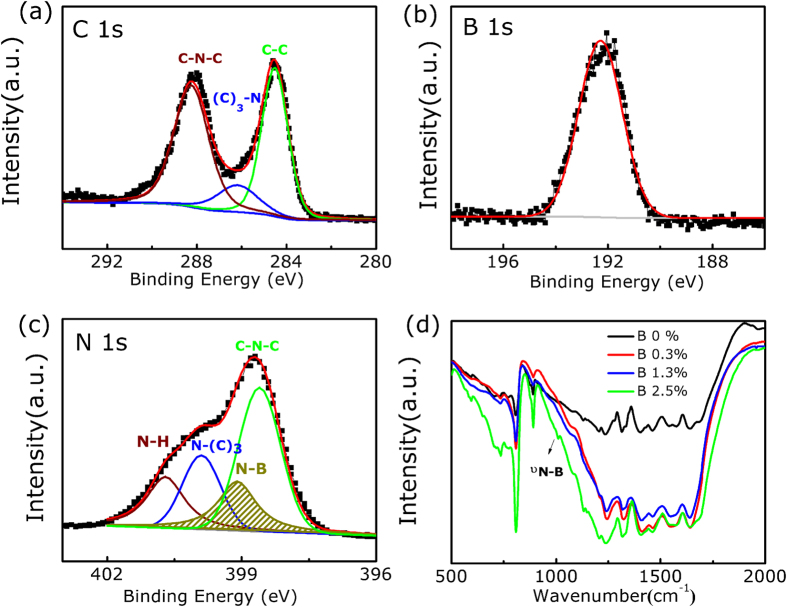
The high resolution XPS spectrum for sample B doped g-C_3_N_4_ nanosheets. (**a**) C 1s (**b**) B 1s, and (**c**) N 1s. (**d**) FTIR spectrum for g-C_3_N_4_ nanosheets with different B concentrations.

In order to determine the oxidation state of B dopant in B-doped g-C_3_N_4_ nanosheets, we examined the X-ray photoelectron spectroscopy (XPS) spectra of the samples where the representative XPS spectra of C 1s, B 1s, and N 1s for the selected B doped g-C_3_N_4_ nanosheets are shown in Fig. 2. The B 1s spectrum in Fig. 2b shows the peak at 192.2 eV, which is basically consistent with the reported binding energy of the C-NB group (192.1 eV) for BCN compound, higher than that of h-BN (190.1 eV) and lower than that of B_2_O_3_ (194 eV)^27^. As shown in Fig. 2a, there are three common peaks at binding energies of 284.5, 286.2 and 288.3 eV, assigned as C-C, (C)_3_-N and C-N-C groupsfor the B-doped g-C_3_N_4,_ respectively, which is similar as primitive g-C_3_N_4_. However, for the N 1s spectrum, besides the main signal shows occurrence of C-N-C groups (398.5 eV), tertiary nitrogen N-(C)_3_ groups (400.1 eV) and N-H group (401.2 eV), there is a new peak at 399.2 eV appears, which is the indicative of N-B groups, having been demonstrated in other graphite-like BCN materials^28^,^29^. Further, in the Fourier transform infrared spectrometer (FTIR) spectra shown in Fig. 2d, the doped materials exhibit a new band at 1011 cm^−1^ compared with the unmodified parental g-C_3_N_4_, which can be attributed to the stretching mode of B-N bond. Combined with the XPS analysis, it can be concluded that B atoms replace C atoms in the g-C_3_N_4_ frame in our case. Besides, FTIR results also feature the typical C-N heterocycle stretches in the 1200–1600 cm^−1^ and the breathing mode of the tris-triazine units at 800 cm^−1^, as the unmodified g-C_3_N_4_, which supports the formation of extended network of C-N-C bonds^30^,^31^.

**Figure 3:**
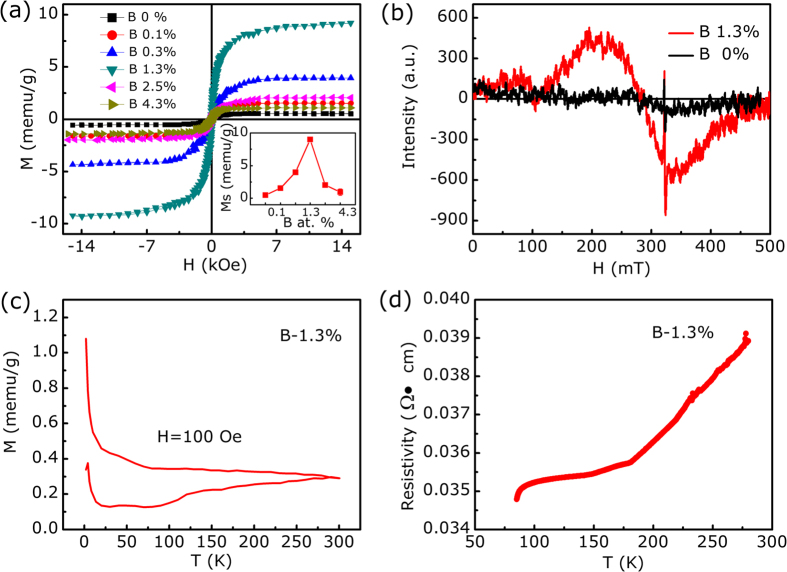
(**a**) M-H curves for g-C_3_N_4_ nanosheets with different B concentration, where the magnetization values were obtained after subtraction of a linear background contribution. Inset shows *M*_s_ dependence on doped B concentration for B doped g-C_3_N_4_ nanosheets. (**b**) ESR results for g-C_3_N_4_ and B doped g-C_3_N_4_ nanosheets (B-1.3%). (**d**) ZFC and FC curves under a measuring field of 100 Oe and (**d**) the temperature dependence of resitivity for B doped g-C_3_N_4_ nanosheets (B-1.3%).

Above results clearly demonstrate that ultrathin B doped g-C_3_N_4_ nanosheets with unique lamellar stacking were successfully obtained. Next, we focus on the magnetic and electrical properties of the B doped g-C_3_N_4_ nanosheets, where we have first eliminated the magnetic impurities by XPS and EELS. To avoid the ferromagnetism contaminations in the preparation progress, all the used tools were plastic^32^. Nonetheless, inductively coupled plasma atomic emission spectroscopy (ICP-AES) was also employed to crosscheck the result. The concentration of *d* or *f* impurities is lower than the detection limit of 10 ppm, which is proved to be not sufficient to produce the obvious ferromagnetic signal. The magnetic properties of the samples were performed with superconducting quantum interference device (SQUID) magnetometry. Figure 3a shows the isothermal magnetization versus applied magnetic field (*M-H*) curves for all the B-C-N samples. As can be seen that, besides the undoped g-C_3_N_4_ nanosheets (which shows the weak ferromagnetism signal of saturation magnetization 0.9 memu/g), all the B doped samples show apparent and well-defined hysteresis loops of diluted magnetic semiconductors after subtraction of linear signal. The inset shown in Fig. 3a indicates that the saturation magnetization (*M*_*s*_) of the samples is B concentration dependence, where the sample of B-1.3% has the maximize *M*_*s*_ of 8 memu/g, similar to that reported in other graphene-related materials^20^,^21^. Besides B doping, results indicate the ferromagnetism of the samples can also be tuned by changing the precursor (Figs S2–S4). Electron spin resonance (ESR) results shown in Fig. 3b give further evidence for the ferromagnetism of the B doped g-C_3_N_4_ nanosheets. It can be seen that the undoped g-C_3_N_4_ nanosheets show the weak center magnetic fields (*H*_*center*_) at 321 mT, which is corresponding to the paramagnetic resonance; however, for the sample B-1.3%, besides the paramagnetic resonance, there is a new *H*_*center*_ at 285 mT appeared, indicating the obvious ferromagnetism of the sample^33^,^34^. Figure 3c show the Zero-field-cooled (ZFC) and field-cooled (FC) temperature dependent magnetization curves of the sample B-1.3% in the temperature ranging from 5 to 300 K. Obviously, ZFC and FC curves show distinct difference in the temperature range, revealing Curie temperature of the sample is higher than 300 K. Most importantly, there is no block temperature appearance in ZFC curve, which clearly reveals that there are no ferromagnetic clusters in our sample and provides evidence for intrinsic ferromagnetism of the sample. Further, the electrical transport property of the synthetic B doped g-C_3_N_4_ nanosheets was measured experimentally from the temperature dependence of the resistivity. As shown in Fig. 3d, in the temperature range of 80–290 K, the resistivity of the sample slightly increases when the temperature arises, which indicates the metallic characteristic and reveals the possibility of metallic or half-metallic nature of B doped g-C_3_N_4_ nanosheets^35^,^36^.

**Figure 4:**
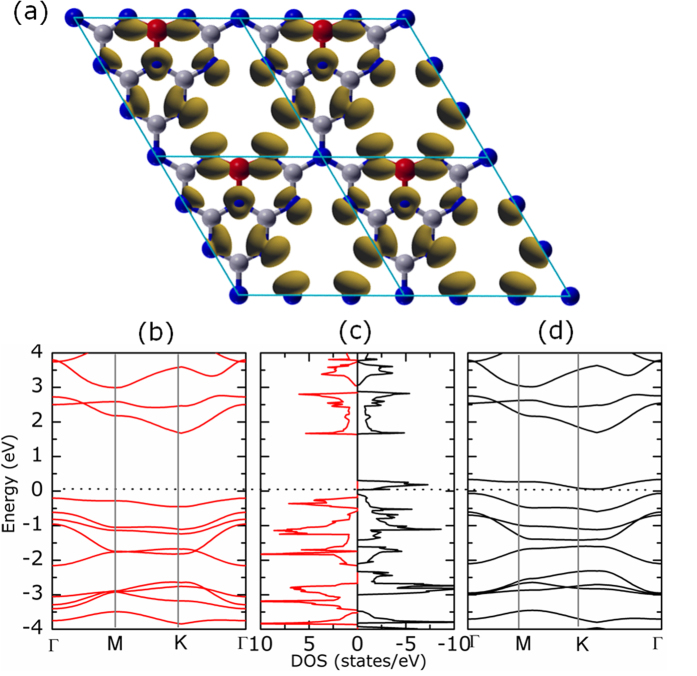
(**a**) The spin density distributions for g-C_3_N_4_ with one C atom replaced by B atom with the contour values of 0.08 and 1.0 Å^−3^, respectively. (**b**–**d**) The corresponding spin-resolved band structure and DOS for g-C_3_N_4_ with one C atom replaced by B atom. The Fermi energy level is taken at 0 eV. The grey, blue and red balls represent carbon, nitrogen and boron atoms, respectively.

To theoretical explore the electronic structure of the synthetic B doped g-C_3_N_4_ nanosheets, we carried out first-principles density functional theory (DFT) calculations. XPS and FTIR results indicate that there are B-N bonds exist in the samples. Further, our previous results indicate that the pure g-C_3_N_4_ nanosheets have nearly no defects (Fig. S2). Therefore, to understand how doped B atoms influence the ferromagnetism and electrical properties in B doped g-C_3_N_4_ nanosheets, single layer g-C_3_N_4_ with one C atom replaced by B atom was used as the calculation model to study the origin of ferromagnetism and metallic or half-metallic nature in carbon nitrides^37^,^38^. The spin density distribution shown in Fig. 4a indicates that the main magnetism originates from the N atoms in this structure and the total magnetic moment is about 1.0 *μ*_*B*_ for one supercell. The dumbbell-shaped spin density distribution reveals that the magnetic moment is mainly attributed to the *p* orbital of N atoms. Figure 4b–d presents the band structure and spin-resolved total density of state (DOS) for a 2 × 2 reconstructed geometry, respectively. As shown in Fig. 4c, a significant asymmetry between the spin-up state and spin-down state in the density of states near the Fermi level suggests the intrinsic ferromagnetism of the B doped g-C_3_N_4_ single layer. Further, it is remarkably that the spin-up channel (red) possesses a very large band gap (1.6 eV), whereas the spin-down one (black) does not show a gap. Thus the charge transport is dominated by the spin-down electron, and the current flow in such a system should be fully spin-polarized, *i*.*e*., half metallicity, which is consistent with our experiment results. Additionally, close examination of the top of the valence band indicates that this receives contributions mainly from the planar *p* atomic orbitals, which is identical with the spin density distribution results^19^. Thus, the inclusion of B doping induced intrinsic ferromagnetism and half metallicity in g-C_3_N_4_ ultrathin nanosheets is verified by theoretical calculations. Experimentally, our results reveal the intrinsic ferromagnetism and metal-like R-T curve (shown in Fig. 3d). For the half-metal property of the sample, observation of spin-polarized current flow is a major claim in experiment. There are some reported references about the measurement of the spin-polarized current such as: spin-polarized current was injected into high Tc superconductors to reduce its critical current^39^, injection and detection of 90% spin-polarized current in GaAs/AlGaAs light-emitting diode^40^, direct-current-switched magnetic memory devices^41^, spin-polarized transport devices^42^. It’s a pity that we didn’t get the similar results in our B doped g-C_3_N_4_ because our samples are powders. And our experiment proofs of observation of spin-polarized current flow in B doped g-C_3_N_4_ are on the way.

**Figure 5:**
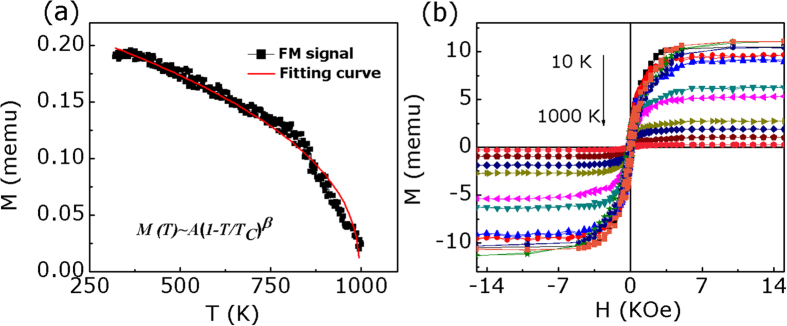
(**a**) The FM signals of the B doped g-C_3_N_4_ nanosheets (B-1.3%) after deducting the PM + DM magnetization fitting results and fitting result. (**b**) M-H curves of the B doped g-C_3_N_4_ nanosheets (B-1.3%) measured from 10 K to 1000 K with SQUID and high-temperature VSM.

In recent years, although lots of low-dimensional materials have been theoretically predicted to exhibit half-metallic ferromagnetism, some of these are hard to achieve in experiment. Here, the fabricated ultrathin B doped g-C_3_N_4_ nanosheets with intrinsic half-metallic ferromagnetism should attract more attention to the electronic structures and physical properties of low-dimensional metal free materials and offer a new direction in the search for new half-metallic materials. The intrinsic ferromagnetism in the B doped g-C_3_N_4_ nanosheets may meet the demand for the design of ultrathin, transparent, and flexible spintronic devices in the future. Further, to estimate the possible application temperature (Curie temperature) of the ferromagnetic B doped g-C_3_N_4_ nanosheets, the temperature dependent magnetization (*M-T*) curve is measured in the range from 300 K to 1000 K at 15 k Oe and the result is shown in Fig. S7. As can be seen, the large contribution represents the diamagnetic (DM) background, which is temperature independent. Besides, the magnetization declines gradually with the increasing temperature in a similar inverse relationship at the beginning, then an inflection point appears at about 800 K. These can be explained by the presence of paramagnetism (PM) mixed with a ferromagnetism (FM) phase. In order to separate the FM signal from the total magnetization (PM + DM + FM signals), we firstly acquired the PM + DM magnetization by fitting the *M–H* curves which were measured at different temperatures in a large field region. The remainder curve after deducting the fitted temperature-dependent PM + DM curve is shown in Fig. 5a, which is the FM part. As can be seen, the date can be fitted with the equation of *M*(*T*) = *A*(*1 − T/T*_*C*_)^*β*^, which is common for a ferromagnetic material. In the equation, A is a coefficient related to the spontaneous magnetization, *T*_*C*_ is the ferromagnetic Curie temperature, and *β* is the critical exponent^43^,^44^. The best fitting curve (*T* < *T*_*C*_) is shown by red curve, where the fitted sample’s Cure temperature is about 951 K. What’s more, the sample’s M-H curves measured from 10 to 1000 K by SQUID and high-temperature vibrating sample magnetometer are shown in Fig. 5b. It also can be seen that the sample’s magnetic movement persists to 1000 K, indicating its high *T*_*C*_. Such high *T*_*C*_ ferromagnetism is much higher than that of traditional magnetic semiconductors and dilute magnetic semiconductors, making them promising candidates for application in spintronic devices.

## Conclusion

In summary, we demonstrated that B atom doping could become a strategy to regulate the magnetic and electrical properties of g-C_3_N_4_ nanosheets. B doped g-C_3_N_4_ nanosheets, as a new metal-free ferromagnetic 2D nanomaterial, have been confirmed by experimentally magnetic studies and theoretical DFT calculations. Further, we revealed their possible half-metal properties in theory prediction. Our results provided a new promising material with tunable magnetic and electronic properties toward spintronics and electronics applications in the future.

## Methods

### Samples preparation

B-doped g-C_3_N_4_ were prepared by heating the mixture of melamine and boron oxide in the closed system for preventing sublimation of melamine. In a typical run, different weights of boron oxide powders were dissolved in 20 mL ethanol solution, and then 4 g of melamine was added into the solution. The mixture was dried at 80 °C and then was put into an alumina crucible with a cover and heated to 500 °C in a muffle furnace for 2 h in N_2_ atmosphere. The product was washed by ethanol many times for removing any possible unreacted boron oxide. Finally, the obtained powders were dispersed in water and then ultrasound for about 10 hours. The initial formed suspension was then centrifuged at about 5000 rmp to obtain the B-doped g-C_3_N_4_ nanosheets^45^.

### Measurement

X-ray diffraction (XRD, X’ Pert PRO PHILIPS with Cu Kα radiation) was employed to study the crystal structure. The morphology of the samples were obtained by using the high resolution transmission electron microscopy (HRTEM, TecnaiTM G2 F30, FEI, USA). Atomic force microscopy (AFM) study in the present work was performed by means of Veeco DI Nanoscope Multi Mode V system. X-ray photoelectron spectroscopy (XPS, VG ESCALAB 210) was utilized to determine the bonding characteristics of the samples. The composition was confirmed by an inductively coupled plasma atomic emission spectrometer (ICP, ER/S). The element analyzer (Varoi EL) was employed to study the C/N rations of the samples. The infrared absorption spectra of the samples were conducted with the KBr pellet method on a Fourier transform infrared spectrometer (FTIR; NEXUS 670, Thermo Nicolet Corp., Madison, WI, USA). The measurements of magnetic properties were made using the Quantum Design MPMS magnetometer based on superconducting quantum interference device (SQUID) and vibrating sample magnetometer equipped with a high temperature chamber (VSM, VSM Model EV9, MicroSense, LLC). Electron spin resonance (ESR JEOL, JES-FA300, microwave frequency is 8.984 GHz) measurement was employed to study the resonance field of the samples. The electrical resistivity of the as-obtained sample was measured on a Keithley 4200 station with the computer-controlled four probe technique.

### Calculations details

The electronic properties are performed via VASP code, which is based on density functional theory with the generalized gradient approximation (GGA) of Perdew-Burke-Ernzerhof (PBE) for the exchange-correlation (XC) potential within the projector augmented wave method. The cutoff energy for plane waves is set to be 400 eV, and the vacuum space is at least 15 Å, which is large enough to avoid the interaction between periodic images. A 9 × 9 × 1 Monkhorst-Pack grid is used for the sampling of the Brillouin zone during geometry optimization and a higher 15 × 15 × 1 Monkhorst-Pack grid for self-consistent calculations. All the atoms in the unit cell are allowed to relax, and the convergence of force is set to 0.01 eV/Å. The model of 2 × 2 supercell with one carbon atom replaced by B was used to simulate the electronic and magnetic properties.

## Additional Information

**How to cite this article**: Gao, D. *et al*. Atomically Thin B doped g-C_3_N_4_ Nanosheets: High-Temperature Ferromagnetism and calculated Half-Metallicity. *Sci. Rep.* **6**, 35768; doi: 10.1038/srep35768 (2016).

## Supplementary Material

Supplementary Information
