# Supplementary material for: Atomically Thin B doped g-C3N4 Nanosheets: High-Temperature Ferromagnetism and calculated Half-Metallicity
Source: Sci Rep. 2016 Oct 20;6:35768. doi: 10.1038/srep35768 (PMC5071904; doi:10.1038/srep35768)
Supplement: Supplementary Information [file srep35768-s1.doc]

**Atomically Thin B doped g-C_3_N_4_ Nanosheets: High-Temperature Ferromagnetism and calculated Half-Metallicity**

Daqiang Gao, Yonggang Liu, Peitao Liu, Mingsu Si*, Desheng Xue*

Key Laboratory for Magnetism and Magnetic Materials of MOE, Key Laboratory of Special Function Materials and Structure Design, Ministry of Education, Lanzhou University, Lanzhou 730000, P. R. China. Fax: +86 0931 8914160; Tel: +86 0931 8914160; *E-mail: sims@lzu.edu.cn, xueds@lzu.edu.cn

**The preparation of B-g-C_3_N_4_ ultrathin nanosheets-film:**

The dispersion of B-g-C_3_N_4_ ultrathin nanosheets was vacuum filtrated onto a cellulose membrane with a 0.22 μm pore size, forming a light-yellow homogeneous thin film，where the thickness of the film can be readily controlled by tuning volume and concentration of the filtrated solution. Second, the obtained B-g-C_3_N_4_ thin film on cellulose membrane was pressed onto the glass substrate with the assistance of small amount of ethanol. Then, the film on the glass substrate was immersed in acetone to gradually dissolved the cellulose membrane. After an hour, the acetone was aspirated and the film was immersed with fresh acetone again. As a result, B-g-C_3_N_4_ ultrathin nanosheets-based film with a 1 cm × 1 cm planar size was successfully transferred onto glass substrate with a size of 2 cm × 4 cm. Finally, we jointed the film and the wire with the colloidal silver





**Figure S1**: XRD results for pure and B doped g-C_3_N_4_ nanosheets.







**Figure S2**: XRD and M-H curves for g-C_3_N_4_ nanosheets obtained by precursor of urea (g-C_3_N_4_ with defects) and melamine (perfect-g-C_3_N_4_).

**Figure S3**:TEM images for g-C_3_N_4_ nanosheets obtained by precursor of urea (g-C_3_N_4_ with defects) and melamine (perfect-g-C_3_N_4_).





**Figure S4**: (a) *M-H* curves for undoped and B doped g-C_3_N_4_ nanosheets obtained by precursor of urea and melamine. (b) variation of *M*_S_ and C/N ration for the four samples.

As can be seen from figure S4, there are C deficiency in the pure g-C_3_N_4_ nanosheets obtained by the precursor of urea (shows the *M*_s_ of 0.0045 emu/g), while, the C/N ration is close to 0.75 for the pure g-C_3_N_4_ nanosheets obtained by the precursor of melamine (shows the *M*_s_ of 0.0009 emu/g), indicating that C defects can introduce the ferromagnetism in the pure g-C_3_N_4_.

Besides defects, doping also can introduce the ferromagnetism in the perfect g-C_3_N_4_ obtained by the precursor of melamine. As can be seen that the sample's *M*_s_ increases to 0.009 emu/g after B doping with the C/N ration of 0.738, which is close to 0.75.

If we introduce both the defects and the B atom doping, the max *M*_s_ is therefore expected. As can be seen, after B doping for the defects existed g-C_3_N_4_, the *M*_s_ of 0.0122 emu/g was obtained, giving the evidence of the introduction of ferromagnetism both by defects and B atom doping.


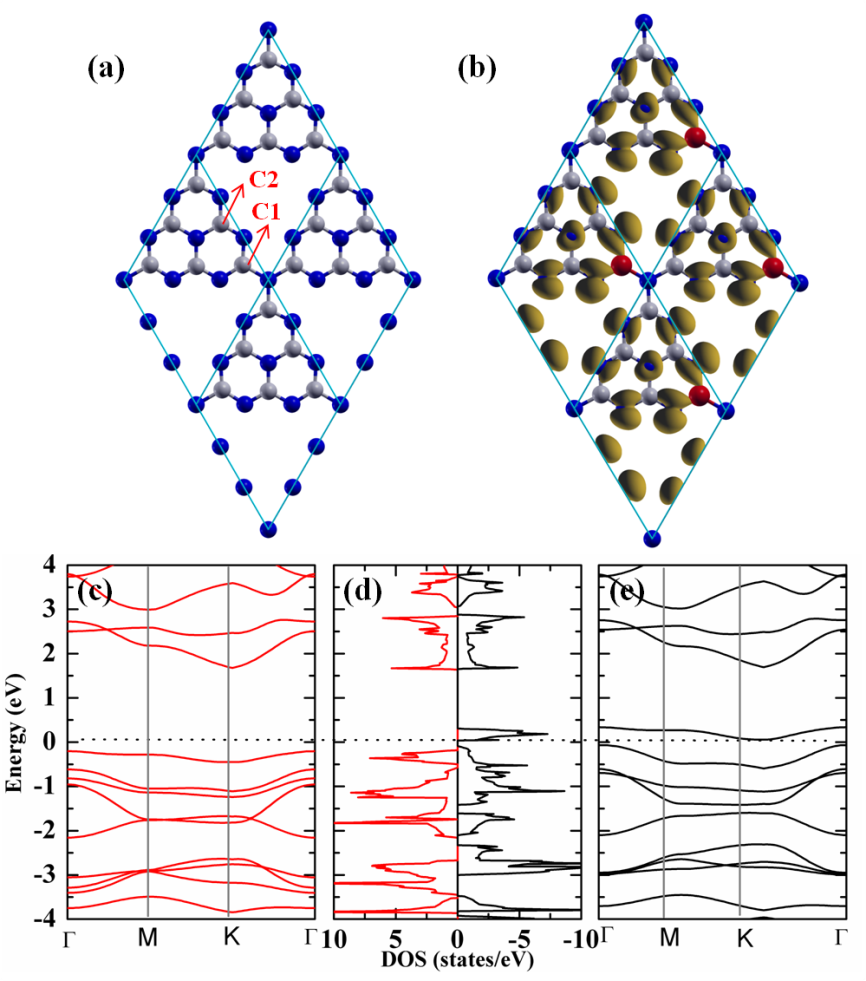


**Figure S5**: (a) The theoretically perfect crystal structure of the single-layered g-C_3_N_4_ with 2×2 supercell, where C atoms have two energetically favored configurations. (b) The spin density distributions for g-C_3_N_4_ with one C atom replaced by B atom at C1 site with the contour values of 0.08 and 1.0 Å ^-3^, respectively. (c-e) The corresponding spin-resolved band structure and DOS for g-C_3_N_4_ with one C atom replaced by B atom. The Fermi energy level is taken at 0 eV. The grey, blue and red balls represent carbon, nitrogen and boron atoms, respectively.

As can be seen form figure S5, there are two different energetically favored configurations in one g-C_3_N_4_ supercell. Results indicate that both one B atom replaces one C atom at C1 and C2 site can introduce spin polarization and the sample will show ferromagnetism and half-metallicity.





**Figure S6**: The magnetic signal of the sample holder dependence on the measured temperature.

**Figure S7**:M-T curves of B doped g-C_3_N_4_ nanosheets (B at. 1.3%). Blue diamonds in the figure represent PM + DM magnetization obtained by fitting the M-H curve measured at that temperature. The red line is the PM + DM magnetization fitting result.
